# Feasibility of synthetic computed tomography generated with an adversarial network for multi-sequence magnetic resonance-based brain radiotherapy

**DOI:** 10.1093/jrr/rrz063

**Published:** 2019-12-10

**Authors:** Yuhei Koike, Yuichi Akino, Iori Sumida, Hiroya Shiomi, Hirokazu Mizuno, Masashi Yagi, Fumiaki Isohashi, Yuji Seo, Osamu Suzuki, Kazuhiko Ogawa

**Affiliations:** 1 Department of Radiation Oncology, Osaka University Graduate School of Medicine, Osaka, Japan; 2 Oncology Center, Osaka University Hospital, Osaka, Japan; 3 Miyakojima IGRT Clinic, Osaka, Japan; 4 Department of Medical Physics and Engineering, Osaka University Graduate School of Medicine, Osaka, Japan; 5 Department of Carbon Ion Radiotherapy, Osaka University Graduate School of Medicine, Osaka, Japan

**Keywords:** synthetic computed tomography, deep learning, generative adversarial network, dose calculation, treatment planning

## Abstract

The aim of this work is to generate synthetic computed tomography (sCT) images from multi-sequence magnetic resonance (MR) images using an adversarial network and to assess the feasibility of sCT-based treatment planning for brain radiotherapy. Datasets for 15 patients with glioblastoma were selected and 580 pairs of CT and MR images were used. T1-weighted, T2-weighted and fluid-attenuated inversion recovery MR sequences were combined to create a three-channel image as input data. A conditional generative adversarial network (cGAN) was trained using image patches. The image quality was evaluated using voxel-wise mean absolute errors (MAEs) of the CT number. For the dosimetric evaluation, 3D conformal radiotherapy (3D-CRT) and volumetric modulated arc therapy (VMAT) plans were generated using the original CT set and recalculated using the sCT images. The isocenter dose and dose–volume parameters were compared for 3D-CRT and VMAT plans, respectively. The equivalent path length was also compared. The mean MAEs for the whole body, soft tissue and bone region were 108.1 ± 24.0, 38.9 ± 10.7 and 366.2 ± 62.0 hounsfield unit, respectively. The dosimetric evaluation revealed no significant difference in the isocenter dose for 3D-CRT plans. The differences in the dose received by 2% of the volume (*D*_2%_), *D*_50%_ and *D*_98%_ relative to the prescribed dose were <1.0%. The overall equivalent path length was shorter than that for real CT by 0.6 ± 1.9 mm. A treatment planning study using generated sCT detected only small, clinically negligible differences. These findings demonstrated the feasibility of generating sCT images for MR-only radiotherapy from multi-sequence MR images using cGAN.

## INTRODUCTION

Magnetic resonance imaging (MRI) is used for the delineation of the organs in radiotherapy because its soft-tissue contrast is superior to that of computed tomography (CT). It has been recommended that MRI should be used for contouring in brain radiotherapy, especially for glioblastoma tumors [1–3]. However, the MR image does not correlate with electron density, and its geometrical accuracy is inferior to that of CT, so MR images are usually registered to reference CT images and the CT images are used for the dose calculation. However, the low contrast of soft tissues on CT images can result in considerable inter-observer variability in volume delineation [4–6], and image registration may lead to additional uncertainty with contouring [7,8]. These issues can be reduced in treatment planning by using MRI only. As additional benefits, the entire planning workload is reduced, there is no need for X-ray irradiation, and direct delineation on MR images allows the margin size to be minimized, thereby reducing toxicity to healthy organs.

The challenge for MR-based radiotherapy planning is to associate the CT number and electron density information with the MR image for dose calculation. To address this issue, several approaches to generate synthetic CT (sCT) images from MR images have been proposed. These include bulk density assignment [9–13], the atlas-based technique [14–17] and the voxel-based method [18–21].

An emerging approach to convert MR to CT images is the application of deep learning using a convolutional neural network (CNN) and a generative adversarial network (GAN) [22]. Han proposed the CNN-based method to generate sCT from T1-weighted (T1w) MRI sequences using U-Net architecture and reported that the CNN-based method performed better than the atlas-based method [23]. However, a limitation of CNN is that it may lead to blurry results due to misalignment between MR and CT [24]. GAN, which is trained by two competing networks, has been applied to MR-to-CT translation. Using adversarial loss, the GAN model can generate high-quality sCT images with less blurry results [24–26]. Emami *et al*. proposed a method of generating sCT from T1w MR using GAN and compared it with the CNN-based method using the same patient cohort [27]. They reported that the GAN-based method preserved anatomical details and improved the quality of sCT images as compared to the CNN approach. However, the primary aim of those studies was to generate sCT images using deep learning, although the use of sCT for radiotherapy has received little attention. Several recent studies [28–32] have investigated sCT-based treatment planning using the CNN or GAN models; however, most of them used single-sequence MR images of the pelvic area. For brain radiotherapy, Dinkila *et al*. generated sCT images from T1w MR images using a 2.5-dimensional convolutional network, which provided acceptable dosimetric results [33]. However, the feasibility of an adversarial network-based sCT generation from multi-sequence MR images with dosimetric evaluation for brain radiotherapy remains uncertain.

Here, we describe a method to generate sCT images from T1w, T2-weighted (T2w) and fluid-attenuated inversion recovery (FLAIR) MR images using a conditional GAN (cGAN) model, and investigate its feasibility for dose calculations in brain radiotherapy.

## MATERIALS AND METHODS

### Patient data and target volume

Datasets that included CT and MR (T1w, T2w and FLAIR) images were retrieved from a freely available database provided by The Cancer Imaging Archive (TCIA) [34]. In total, 580 pairs of CT and MR images of 15 patients were collected. All CT images were acquired with GE Medical Systems scanners with a median resolution of 0.49 × 0.49 × 2.5 mm^3^ (range, 0.45–0.56 × 0.45–0.56 × 1.25–5.0 mm^3^). The matrix size was 512 × 512, with a median field-of-view (FOV) of 25 cm (range, 23–28 cm). For the MR images, the median voxel size was 0.45 × 0.45 × 5.0 mm^3^ (range, 0.43–0.94 × 0.43–0.94 × 0.90–5.0 mm^3^). The sequences included images acquired with Philips Medical Systems, GE Medical Systems and Siemens scanners. The median FOV was 23 cm for all sequences.

The gross tumor volumes (GTV) were contoured using registered MR images. GTV1 was identified from the T1w image and GTV2 from the T2w or FLAIR image by a single observer, which was then reviewed by an experienced radiation oncologist. The clinical target volumes (CTV) were defined as the GTVs plus a 1.5-cm margin within the brain. The planning target volumes (PTV) were defined as the CTVs plus a 0.5-cm margin. The prescribed doses to PTV1 and PTV2 were 60 and 40 Gy, respectively, delivered in 30 fractions.

### Image preprocessing

The field inhomogeneities of MRI were corrected using the N4 bias field correction algorithm [35]. The coordinates were aligned between the CT and MR images by applying deformable image registration using the SmartAdapt application of the Eclipse version 15.1 treatment planning system (TPS) (Varian Medical Systems Inc., Palo Alto, CA). The pixel intensities of the MR images were normalized to values in the range 0–4095 using the method proposed by Nyul and Udupa [36]. To separate the patient body from the background, we used the binary mask by applying the Gaussian filter, the Otsu threshold method [37] and the morphology operation. The background voxels of the CT images were filled with −1000 and those in the MR images with 0. So that the MR image of all sequences could be input to the network as a single image, the T1w, T2w and FLAIR images of the same slice were combined into three channels in a color image.

### Network architecture

**Fig. 1:**
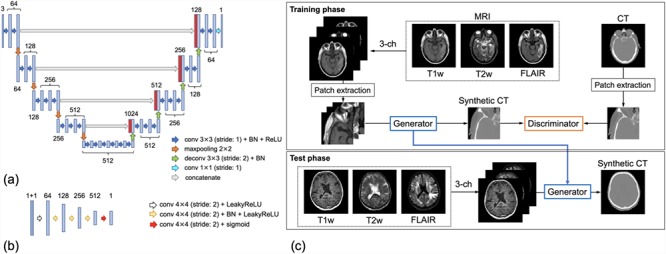
Schematic representation of the proposed method to generate sCT images. (**a**) The architecture of the generator and (**b**) discriminator. (**c**) Overview of the cGAN-based method to generate sCT images from multiple MR sequences. BN = batch normalization; conv = convolutional layer; deconv = deconvolutional layer; LeakyReLU = leaky rectified linear units; ReLU = rectified linear units; sigmoid = sigmoid activation function.

A cGAN-based network [38,39] was used to generate sCT images from multi-sequence MR images. Fig. 1 illustrates the workflow of our proposed method for generating sCT images from multiple MR sequences. The architecture of the generator and discriminator is shown in Fig. 1a and b, respectively. For the generator, we used the U-Net architecture [40] proposed by Fu *et al*. [41], which was modified to handle color images and to fit to our datasets. The discriminator was based on the PatchGAN discriminator used in the pix2pix model.

### Training details

Patch-based training was performed because of the limited number of medical images and the limited memory of the graphics processing unit (GPU). Patches of 128 × 128 were extracted from the MR and corresponding CT images for use as training data. The weights of the generator and discriminator were initialized by He initialization [42] and the biases were initialized to zero. As proposed by Isola *et al*. [39], the loss function by L1 norm of the generator was the mean absolute error (MAE) between the real CT (rCT) image and the sCT image as follows:

(1) \begin{equation*} MAE=\frac{1}{n}\sum_{i=1}^n\left|{rCT}_i-{sCT}_i\right|, \end{equation*}

where *n* is the number of voxels, and rCT*_i_* and sCT*_i_* represent the CT numbers in the *i*-th voxels of the rCT and sCT images, respectively.

The patch size of the discriminator was 32 × 32. The network was evaluated with the 5-fold cross-validation method, where 15 datasets were randomly divided into five groups. For each fold, the model was trained using four of the datasets and the sCT was predicted from the fifth dataset using the trained model. This process was repeated for the five groups. In the training phase, the training data were divided for training and validation at a ratio of 9:1. Data augmentation was performed using random horizontal flips, image shifts, zooms and rotations. Training was performed using an NVIDIA GTX 1080 Ti (11 GB) GPU with a mini-batch of 32.

**Fig. 2:**
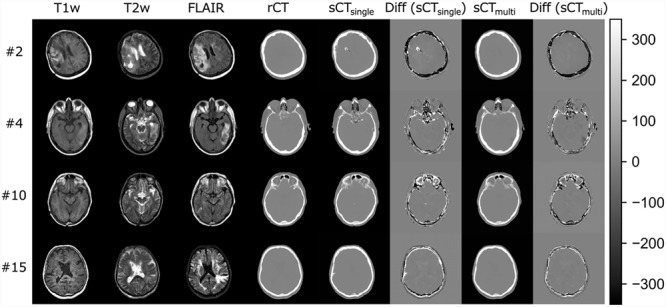
Quantitative comparisons of axial slices between the real and synthetic CT images (rCT and sCT, respectively). Each row shows images of a different representative patient. The sCT images generated from only the T1w sequence (sCT_single_) and from the T1w, T2w and FLAIR MR sequences (sCT_multi_) are shown. The difference maps show the differences in CT number between the rCT and sCT images (sCT—rCT) according to the color bar on the right.

### Synthetic CT generation

sCT images were generated from full-sized MR images (512 × 512) using the trained generator with the GPU calculation, even though training was performed using patch images (128 × 128) as shown in Fig. 1c. The datasets were not created for use in radiotherapy, so the full MR volume corresponding to the registered CT was not obtained. As a result, the generated sCT image lacked the top or bottom slices, as compared to the rCT volume. This issue was compensated by filling the missing slices with the corresponding slices of the rCT and replacing the CT numbers with values calculated from the correlation between the predicted and original voxel values. In particular, we replaced the original CT number in the missing regions with the median of all the predicted hounsfield unit (HU) values, which were converted from all voxels with the corresponding CT number. For the purposes of comparison, sCT images were also generated from only a single T1w sequence. Thus, sCT_single_ was defined as sCT generated from a single T1w MR image and sCT_multi_ from multiple MR sequences (T1w, T2w and FLAIR). The networks for sCT_single_ and sCT_multi_ were trained separately.

### Evaluation of image quality

Image quality was evaluated by calculating the voxel-wise MAE between the sCT and rCT images with respect to the CT number. The MAE of the CT number was evaluated for the entire body region, for soft tissue and for bone regions. The soft tissue and bone region were extracted by setting CT number thresholds of −100 to 150 HU and ≥250 HU, respectively.

### Treatment planning

The sCT-based dose calculation was evaluated by applying 3D conformal radiotherapy (3D-CRT) and volumetric modulated arc therapy (VMAT) techniques. The treatment plans were calculated with the Eclipse TPS. In the sCT plans, the body was delineated using the sCT images and the other structures were copied from those of the rCT.

For 3D-CRT evaluation, boost plans up to 40–60 Gy were compared to clarify the impact of using the sCT for the dose calculation. A dose of 20 Gy in 10 fractions was prescribed to the isocenter to cover the PTV1 at a dose of 95% using the anisotropic analytical algorithm (version 13.7.14) of the Eclipse TPS.

The simultaneous integrated boost technique was used for VMAT evaluation. Single-arc VMAT plans were created with a collimator angle of 30°. The VMAT plans were calculated using the Acuros XB algorithm (version 15.1.51) with a 6-MV photon beam and were optimized by applying the constraints reported by the Radiation Therapy Oncology Group (RTOG 0825) [43]. The VMAT plans were normalized to 95% of the prescribed dose to PTV1. An sCT-based plan was created by recalculating the rCT plan with the sCT images, with the same multileaf collimator sequence and monitor unit as calculated in the rCT. A calculation grid size of 2.5 mm was used in both algorithms.

Dose differences were evaluated with respect to the isocenter dose for the 3D-CRT and dose–volume parameters for the VMAT. In particular, we compared the dose received by 2% of the volume (*D*_2%_), *D*_50%_ and *D*_98%_, the volume receiving at least 95% of the prescribed dose (*V*_95%_) for PTV1, the mean dose for the body, and the maximum dose (*D*_max_) for the organs at risk.

The impact of sCT on the equivalent path length (EPL) was also evaluated. Assuming a single-arc VMAT, EPLs were compared between the rCT and sCT for 180 beams at angles from 181°–179° at intervals of 2°.

The dose distribution agreement with the original plan was compared by the 2D gamma evaluation [44] with the criteria of 3%/3 mm, 2%/2 mm and 1%/1 mm for the dose difference and the distance.

### Statistical analysis

All statistical analyses were performed using JMP Software (SAS Institute Inc., Cary, NC). Depending on the normality of the data distributions, dose differences between the rCT and sCT plans were compared using either the paired *t*-test or the Wilcoxon signed-rank test. Statistical significance was defined as *P* < 0.05.

## RESULTS

### Image quality evaluation

Each training session of 500 epochs took ~2.5 days. For testing, sCT images were predicted using the trained network in 17.4 ± 5.4 s per patient.

**Fig. 3:**
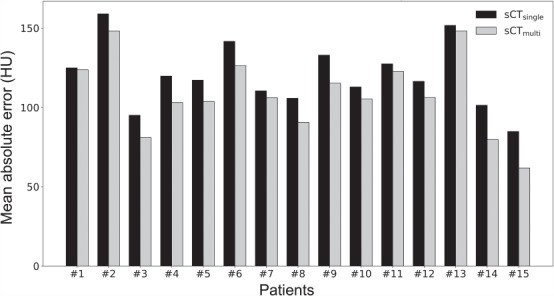
A comparison of MAE within the body contour between sCT_single_ and sCT_multi_ of all 15 patients.

Fig. 2 shows difference maps between the sCT and rCT images for representative patient datasets. The MAE values of sCT_single_ and sCT_multi_ for the whole body are shown in Fig. 3. The MAE values of sCT_multi_ were lower than those of sCT_single_ in all patients. The mean ± standard deviation (SD) MAE values for the CT numbers for the whole body, soft tissue and bone regions are summarized in Table 1. The overall MAE values of sCT_multi_ were significantly smaller than sCT_single_. The proposed approach to use multiple MR sequences was found to generate better quality sCT images, similar to rCT rather than sCT_single_.

**Fig. 4:**
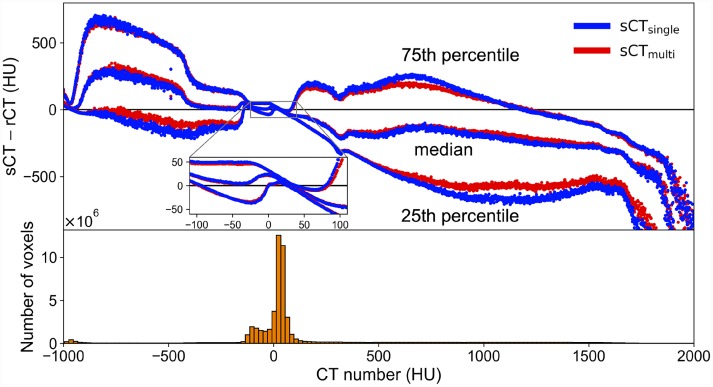
The difference in CT numbers between the synthetic and real CT images (sCT and rCT, respectively). The median, 25th and 75th percentiles of absolute differences (sCT—rCT) are shown with a histogram of the distribution of the voxels inside the patient body contour (bin size, 20). The horizontal axis indicates the rCT CT numbers and the upper vertical axis indicates the absolute difference in CT number. The inset expands the region between −100 and 100 HU, which included more than 70% of all the voxels. In this region, the median differences were within ±50 HU.

Fig. 4 shows how the difference in CT numbers between the sCT and rCT varied with the CT number for all datasets. Data for voxels >2000 HU are not displayed because the ratio to whole voxels was <0.05%. The median differences for voxels between −100 and 100 HU (which included more than 70% of all the voxels) were within ±50 HU. In the positive CT number region, the prediction errors of the sCT_multi_ images were smaller than those of the sCT_single_ images. In addition, the range from the 25th to 75th percentile of sCT_multi_ was smaller than that of sCT_single_.

**Table 1:** The MAE of CT number between the sCT_single_ and sCT_multi_ images

	Mean ± SD (HU)		
	sCT_single_	sCT_multi_	*P*-value
Body	120.1 ± 20.4	108.1 ± 24.0	<0.001
Soft tissue region (−100 HU<, <150 HU)	46.3 ± 9.3	38.9 ± 10.7	<0.001
Bone region (≥250 HU)	399.4 ± 51.8	366.2 ± 62.0	<0.001

### Dosimetric evaluation

**Fig. 5:**
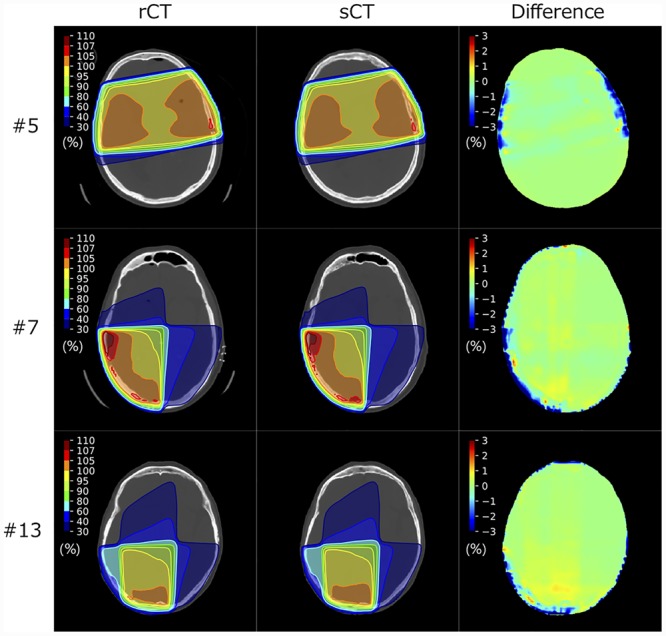
Dose distributions of sCT_multi_ of representative 3D-CRT plans and associated difference maps showing the dose difference relative to the prescribed dose of 20 Gy.

**Fig. 6:**
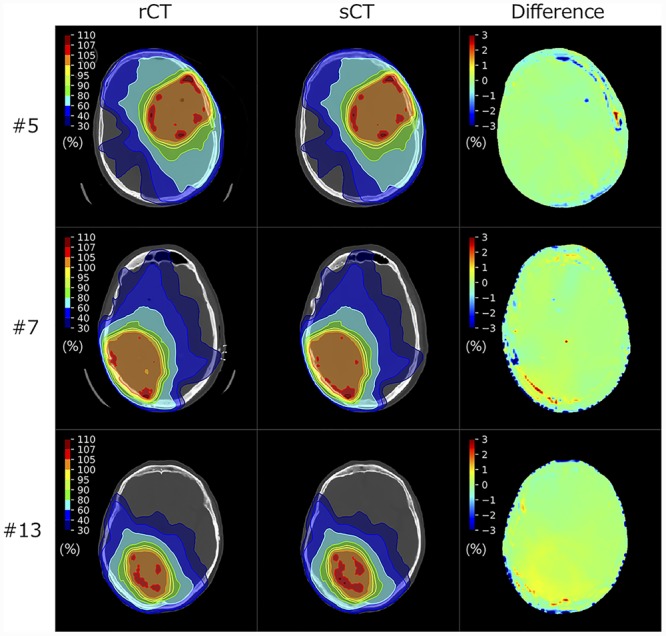
Dose distributions of sCT_multi_ of representative VMAT plans and associated difference maps showing the dose difference relative to the prescribed dose of 60 Gy. Each row shows the same slices as in Fig. 5.

In the 3D-CRT plans, the mean ± SD isocenter dose for the sCT_multi_ plans was 2003.3 ± 8.0 cGy, which was not significantly different from the reference dose of 2000.0 cGy in the rCT plans (*P* = 0.14). Representative dose distributions of sCT_multi_ for the 3D-CRT and VMAT plans are shown in Figs. 5 and 6.

**Table 2:** Comparison of dose–volume parameters between the sCT_multi_ and rCT plans. The data show the mean dose–volume parameters for each structure and the differences relative to the prescribed dose of 60 Gy. *D*_*x*%_ indicates the dose that covered *x*% of the volume. *V*_*x*%_ indicates the volume that received *x*% of the prescribed dose.

Structures	Parameters	Mean ± SD		Difference (%)	*P*-value
		rCT	sCT_multi_		
PTV1	*D*_2%_ (Gy)	64.0 ± 0.5	64.4 ± 0.6	0.68	0.008
	*D*_50%_ (Gy)	61.9 ± 0.3	62.1 ± 0.4	0.34	0.011
	*D*_98%_ (Gy)	59.4 ± 0.2	59.6 ± 0.3	0.30	0.010
	*V*_95%_ (%)	100.0 ± 0.1	100.0 ± 0.1	0.01	0.064
Body	*D*_mean_ (Gy)	19.7 ± 6.2	19.8 ± 6.3	0.09	0.14
Brainstem	*D*_max_ (Gy)	46.4 ± 16.6	46.6 ± 16.7	0.35	0.018
Optic chiasm	*D*_max_ (Gy)	33.0 ± 21.8	33.1 ± 21.8	0.20	0.004
Optic nerve (left)	*D*_max_ (Gy)	22.0 ± 18.8	22.1 ± 18.9	0.23	0.027
Optic nerve (right)	*D*_max_ (Gy)	23.1 ± 20.0	23.2 ± 19.9	0.04	0.59
Eye (left)	*D*_max_ (Gy)	20.0 ± 15.0	20.0 ± 15.1	0.10	0.42
Eye (right)	*D*_max_ (Gy)	21.2 ± 15.8	21.3 ± 16.1	0.27	0.068
Lens (left)	*D*_max_ (Gy)	6.1 ± 4.1	6.1 ± 4.1	0.00	1
Lens (right)	*D*_max_ (Gy)	6.0 ± 4.5	6.0 ± 4.5	0.00	1

Table 2 compares the dose–volume parameters between rCT and sCT_multi_ for the VMAT plans. The dose parameters tended to be higher in the sCT_multi_ plans. The target coverage (*V*_95%_) of the sCT_multi_ plan was preserved, with no significant difference. *D*_2%_, *D*_50%_ and *D*_98%_ for PTV1, and *D*_max_ for the brainstem, optic chiasm and left optic nerve were significantly higher with the sCT_multi_ than the rCT. Dose differences relative to the prescribed dose were within 1.0% for all parameters.

### Equivalent path length

**Fig. 7:**
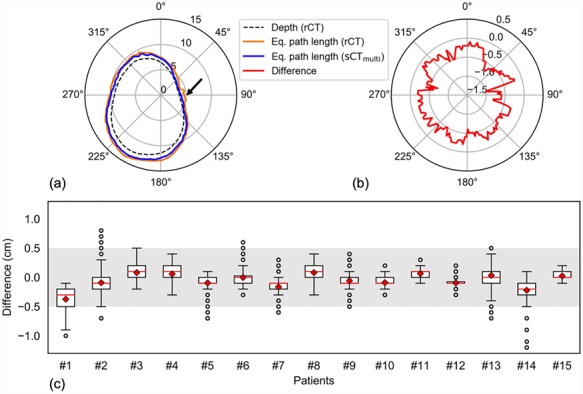
Comparison of the EPLs between the real and synthetic CT (rCT and sCT, respectively) plans, where the latter was generated from multiple MR sequences plans. (**a**) A representative example of the EPL (for Patient #1). The dashed line indicates the physical depth of rCT (in cm) and the solid lines indicate the EPL (in cm) for rCT and sCT_multi_. The arrow indicates the implantation of metal. (**b**) Differences in EPL between the sCT_multi_ and rCT plans (sCT_multi_—rCT). (**c**) Boxplot of the EPL differences for all 15 patients. The diamonds denote the mean values and the gray area indicates the region with differences between −0.5 and 0.5 cm.

The mean ± SD EPL error for the sCT_multi_ was −0.6 ± 1.9 mm (range, −12.0 to 8.0 mm) compared with that for the rCT. An example of the difference in EPL is illustrated in Fig. 7a and b. Fig. 7c shows the variation in the difference of EPL (sCT—rCT) for each patient. The EPLs with errors between −5.0 and 5.0 mm accounted for >98% of all beams for whole datasets. A large error was found in the metal placement (Fig. 7a).

### Dosimetric comparison of sCT_single_ and sCT_multi_

**Fig. 8:**
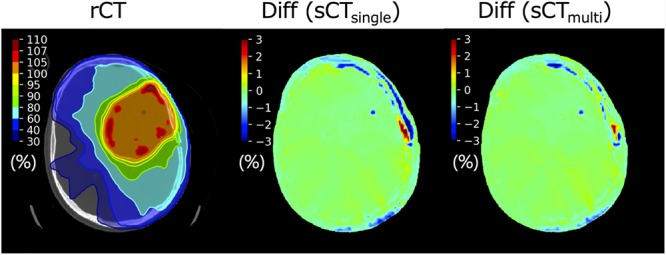
Comparison of the dose difference maps for VMAT plans between sCT_single_ and sCT_multi_ (Patient #5).

The dose difference maps of sCT_single_ and sCT_multi_ of a representative patient are shown in Fig. 8. Dose errors around the left temporal bone were improved for the sCT_multi_, as compared with sCT_single_.

The gamma evaluation results are summarized in Table 3. Although the pass rates for both sCT_single_ and sCT_multi_ were >94% even with the 1%/1 mm criterion, the pass rates of sCT_multi_ were significantly higher than those of sCT_single_ with all criteria. For EPL evaluation, the mean EPLs of sCT_single_ and sCT_multi_ were −0.5 ± 2.1 mm (range, −16.0 to 10.0 mm) and −0.6 ± 1.9 mm (range, −12.0 to 8.0 mm), respectively. Although the effects of the MR sequences on the dose-volume histogram (DVH) parameters of the target and organs at risk were <1% (data not shown), multi-sequence MR improved the accuracy of sCT generations in terms of CT values and dose calculations, particularly in the bone region.

**Table 3:** Gamma pass rates (%) of the sCT_single_ and sCT_multi_

Criterion	Mean ± SD (range)		*P*-value
	sCT_single_	sCT_multi_	
3%/3 mm	99.7 ± 0.5 (98.3–100.0)	99.8 ± 0.3 (99.1–100.0)	0.045
2%/2 mm	98.9 ± 1.2 (96.0–99.8)	99.2 ± 1.0 (97.1–100.0)	0.004
1%/1 mm	94.2 ± 4.9 (83.4–98.6)	95.3 ± 4.7 (85.0–99.0)	0.002

## DISCUSSION

In this study, sCT images were generated from multi-channel MR images using a cGAN model. The input was multi-sequence T1w, T2w and FLAIR MR images combined into a single-input image with three channels. The images were used to simulate sCT-based treatment planning and the resulting dose distributions were compared with the ground truth rCT doses. The results showed that our proposed approach was able to generate sCT images that provided dose distributions similar to those of the reference images. To the best of our knowledge, this is the first report to describe the use of multi-sequence brain MR images to create sCT images and then to investigate the impact of using the sCT images for the dose calculation. Maspero *et al*. generated sCT images using three MRI sequences, including in-phase, fat and water MR images, of the pelvic region [32]. Their sampling size for sCT generation was 256 × 256 of 8-bit CT images, whereas our method used 512 × 512 samplings of 16-bit images, providing better image quality.

sCT for the head region has been reported to have higher MAEs (80–200 HU) when compared with those for the pelvic region, in most cases because of the complex structures and the high ratios of air and bone [28, 45]. Our MAE results were superior or comparable to those of the atlas-based [17] and classification-based results [18, 20, 46]. Although Andreasen *et al*. achieved a MAE of 85 HU using the patch-based method, it took 15 h to generate sCTs from the T1w images [47]. Uh *et al*. used a multi-atlas approach to generate sCTs from MRI, with their proposed method taking 120.6 and 271.4 min using 6 and 12 atlases, respectively [48]. Our proposed method generated sCT images within 20 s per patient. The major advantage of the deep-learning-based method is the fast computation time for predicting sCT images, although the network must be re-trained when training datasets are updated or when input data are changed. For the deep-learning-based methods, Han generated sCT images from single-sequence (T1w) MR images using a deep CNN and reported a MAE of 84.8 ± 17.3 HU [23]. Emami *et al*. compared CNN and GAN models for the generation of sCT images and reported that the MAE of the GAN model was better than that of the CNN model (89.3 ± 10.3 vs 102.4 ± 11.1 HU, respectively) [27]. A 3D GAN model by Nie *et al*. achieved a MAE of 73.7 ± 2.3 HU [26]. Recently, the cycle GAN-based method proposed by Lei *et al*. yielded a promising MAE of 55.7 HU for brain patients using single T1w sequence images [25]. The MAE of sCT_multi_ was 108.1 ± 24.0 HU, which was slightly higher than the previous results for the head site, although for a different patient population. However, when comparing the MAE values between sCT_multi_ and sCT_single_ for each patient, sCT_multi_ values were lower in all patients. In addition, our proposed approach demonstrated superior accuracy in predicting sCT in terms of maintaining the structural details. As shown in Fig. 2, low-intensity voxels within the brain of T1w images were predicted as air or bones in sCT_single_ images. In contrast, they were accurately assigned to the appropriate tissues in sCT_multi_ images; this is another advantage of using multiple sequences to generate sCT from MR images. One reason for the relatively larger MAE than other studies may be due to the prediction errors in regions with high CT numbers. As shown in Fig. 4 (upper), there was a large discrepancy with sCT in the regions with high CT numbers. The datasets used for this work included some patients with postoperative clips. There were only small differences from the true CT number in the majority of voxels, such as those in regions of soft tissue (−200 to 150 HU); however, the large deviation in a few pixels with high CT numbers may have affected the MAE results. This deviation was probably because the number of images that included high CT number materials was too low a proportion of the whole set of training images to allow for accurate prediction. The prediction accuracy of bone-like regions can be improved by increasing the training sample size.

In contrast, the mean error for the EPLs was −0.6 ± 1.9 mm for sCT_multi_. The impact on dose calculations of such a small difference in EPLs would be negligible. The comparison of 3D-CRT plans showed that the difference in isocenter dose between the rCT and sCT_multi_ images was within 0.2%, which might be attributed to the small proportion of bone relative to the whole beam path for a brain site in spite of a large MAE for the bone regions. Dinkla *et al*. also reported small differences in the EPLs and dose calculations for the brain, although the input MR sequence and network structure in their study differed from those used in our study [33]. Paradis *et al*. investigated sCT-based brain VMAT treatment planning using the voxel-based method with fuzzy c-means clustering, achieving mean differences of *D*_5%_, *D*_95%_ and *D*_max_ for PTV of <1.0%, with the largest difference of 0.6 Gy [21]. In our proposed method, the percentage error relative to the prescribed dose was <1.0% and the largest error in *D*_2%_ for PTV1 was an absolute difference of 0.4 Gy; these differences may be acceptable from a clinical viewpoint. However, deviations of CT numbers in bone may lead to large dosimetric errors in regions where the proportion of bony anatomy is higher than that of soft tissue.

The MAE values of the sCT_multi_ images were significantly lower than those of the sCT_single_ images, resulting in larger dose errors around the bone regions (Fig. 8). Consequently, the pass rates for sCT_multi_ were improved compared with those for sCT_single_. These results indicate that the multiple MR sequences generated potentially similar dose distributions as those of the original CT image. Although our proposed method using multi-sequence MR will require additional time and cost in clinical application, the use of multiple sequences provided better results than those provided by a single T1w sequence in the same patient population.

There were some limitations to this study. First, the patient data used in this study were derived from The Cancer Imaging Archive (TCIA) online data. The patients were not actually treated with the treatment protocol of this study. Analysis of the data of patients who received radiotherapy will provide more realistic results. Second, the sCT used for treatment planning included slices that were not predicted using the trained network, in particular at the top and/or bottom of the volume. As described in the Materials and Methods section, the datasets used in this study were not for radiotherapy, so the full MR volume corresponding to CT was not available. The compensation process may have affected the dose–volume histogram data, although there was no effect on the isocenter dose or EPL results. Third, there were additional uncertainties associated with the deformable image registration to match the alignment between the CT and MR images, which was applied three times for each sequence. As reported previously [49, 50], errors between the three sequences may therefore have affected the MAE results. These errors will have less of an impact by applying rigid image registration, rather than deformable image registration, for patients scanned at one time for all sequences.

In conclusion, we developed a technique to generate sCT images from multi-sequence brain MR images using an adversarial network. The performance of the model was evaluated by comparing the image quality and the treatment planning with those of the original CT images. The use of multiple MR sequences for sCT generation using cGAN provided better image quality and dose distribution results compared with those from only a single T1w sequence. The CT number of the generated sCT images showed good agreement with the original images, but not in the bone regions. Impacts on the dose calculations were within 1%. These findings demonstrate the feasibility and utility of sCT-based treatment planning and support the use of deep learning for MR-only radiotherapy.
